# Radiotherapy-induced bone deterioration is exacerbated in diabetic rats treated with streptozotocin

**DOI:** 10.1590/1414-431X2021e11550

**Published:** 2021-10-29

**Authors:** Maogang Jiang, Yuanjun Ding, Shiwei Xu, Xiaoxia Hao, Yongqing Yang, Erping Luo, Da Jing, Zedong Yan, Jing Cai

**Affiliations:** 1 Department of Biomedical Engineering, Fourth Military Medical University, Xi’an, China; 2 Department of Medical Technical Support, NCO School of Army Medical University, Shijiazhuang, China; 3 Laboratory of Tissue Engineering, Faculty of Life Sciences, Northwest University, Xi'an, China; 4 State Key Laboratory of Military Stomatology, Fourth Military Medical University, Xi'an, China; 5 College of Basic Medicine, Shaanxi University of Chinese Medicine, Xianyang, China

**Keywords:** Radiotherapy, Diabetes mellitus (DM), Bone loss, Osteoblasts, Osteocytes

## Abstract

Following radiotherapy, patients have decreased bone mass and increased risk of fragility fractures. Diabetes mellitus (DM) is also reported to have detrimental effects on bone architecture and quality. However, no clinical or experimental study has systematically characterized the bone phenotype of the diabetic patients following radiotherapy. After one month of streptozotocin injection, three-month-old male rats were subjected to focal radiotherapy (8 Gy, twice, at days 1 and 3), and then bone mass, microarchitecture, and turnover as well as bone cell activities were evaluated at 2 months post-irradiation. Micro-computed tomography results demonstrated that DM rats exhibited greater deterioration in trabecular bone mass and microarchitecture following irradiation compared with the damage to bone structure induced by DM or radiotherapy. The serum biochemical, bone histomorphometric, and gene expression assays revealed that DM combined with radiotherapy showed lower bone formation rate, osteoblast number on bone surface, and expression of osteoblast-related markers (ALP, Runx2, Osx, and Col-1) compared with DM or irradiation alone. DM plus irradiation also caused higher bone resorption rate, osteoclast number on bone surface, and expression of osteoclast-specific markers (TRAP, cathepsin K, and calcitonin receptor) than DM or irradiation treatment alone. Moreover, lower osteocyte survival and higher expression of Sost and DKK1 genes (two negative modulators of Wnt signaling) were observed in rats with combined DM and radiotherapy. Together, these findings revealed a higher deterioration of the diabetic skeleton following radiotherapy, and emphasized the clinical importance of health maintenance.

## Introduction

Radiotherapy is the most commonly used approach for cancer treatment after surgery or chemotherapy (1). The high-energy rays can destroy cancer cells by inducing nuclear DNA damage and subsequent cell death, while the health of adjacent normal tissues/cells is also compromised (2). As life expectancy of patients continues to increase with advances in cancer treatment, radiation-induced long-term adverse effects has become a rising concern for clinicians. Bone damage is a common chronic complication associated with radiotherapy (3,4). It has been shown that cancer patients experience progressive bone loss and increased risks of fragility fractures after radiotherapy (5,6). Even worse, increasing clinical trials and case reports have raised the possibility of radiotherapy-induced osteonecrosis (7,8). Substantial animal studies have reported that focal radiation leads to significant loss of bone mass and deterioration of bone microarchitecture and mechanical properties (4,9,10). Furthermore, local radiation has been shown to induce systemic adverse changes in bone volume and microarchitecture (11). Because osteoporotic fractures (especially hip fractures) are associated with increased mortality and morbidity, it is of great clinical importance to fully understand the etiology and pathology of radiation-induced bone damage.

Diabetes mellitus (DM) is a public health issue in which the patient either does not produce enough insulin or does not respond appropriately to insulin, and it is estimated to affect over 400 million people worldwide (12). The global diabetes prevalence will continue to rapidly increase in the following decades, especially in developing countries (13). Patients with DM are prone to damage of many organs (e.g., heart, nervous system, and kidney), and bone injury is also a common clinical complication resulting from DM (14,15). Type 1 diabetic patients have reduced bone mineral density, compromised bone microstructure, and impaired bone mechanical properties (16). The relative risk of hip fracture in type 1 diabetic patients ranges from 7.1 to 11.2 compared with age-matched nondiabetics (17). Similarly, type 2 diabetic patients also have poorer bone quality and higher risk of fragility fractures than normal populations (18). When a fracture occurs in diabetic patients, DM-induced microvascular dysfunction and elevated infection risk delay healing and increase morbidity and mortality (19). However, to our knowledge, no clinical or experimental study has systematically characterized the bone phenotype of the diabetic patient following radiotherapy. A full understanding of the combined effects of DM and radiation on bone status will be of great benefit in maintain the health of diabetic patients subjected to radiotherapy.

Therefore, in the current study, the effects of radiation on non-diabetic and diabetic bone mass and microarchitecture were evaluated via systematic bone micro-computed tomography (micro-CT) analysis in rats treated with streptozotocin. Moreover, the effects of DM, irradiation, and DM combined with irradiation on the biological activities of osteoblasts, osteoclasts, and osteocytes were compared at the cellular level and molecular level based on serum biochemistry, skeletal histomorphometry, and gene expression assays.

## Material and Methods

### Animals and study design

Thirty-two male Sprague-Dawley rats (3 months, 280-350 g) were acquired from the Animal Center of the Fourth Military Medical University. Rats were housed in the laboratory environment at room temperature (24±1°C) and 55±5% relative humidity with a 12-h-light/dark cycle for one week before experiments. Rats were given free access to water and rodent chow pellets.

**Figure 1:**
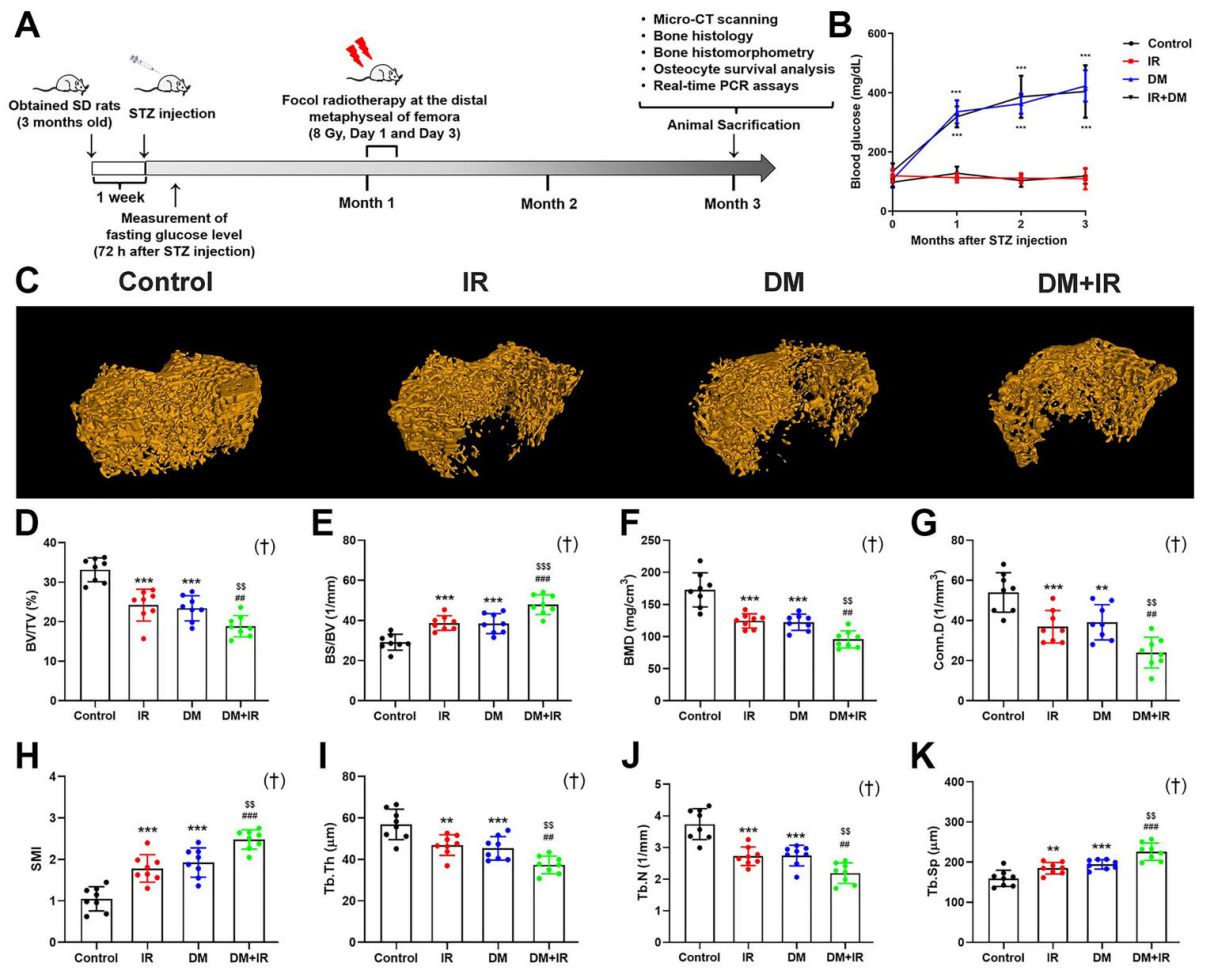
A, The schematic model of the present study. **B**, The fasting blood glucose levels of all rats in the four groups after streptozotocin (STZ) administration. **C**, Representative femoral trabecular bone micro-CT images selected from the volume of interests starting from the proximal end of the growth plate of distal femur and extending 2.5 mm to the proximal end in Control, irradiation (IR), diabetes mellitus (DM), and DM+IR rats. **D**-**K**, Quantitative analysis of micro-CT results, including bone volume/total volume (BV/TV), bone surface/bone volume (BS/BV), bone mineral density (BMD), connectivity density (Conn.D), structure model index (SMI), trabecular thickness (Tb.Th), trabecular number (Tb.N), and trabecular separation (Tb.Sp). Data are reported as means±SD (n=8). **P<0.01, ***P<0.001 *vs* Control group; ^##^P<0.01, ^###^P<0.001 *vs* IR group; ^$$^P<0.01, ^$$$^P<0.001 *vs* DM group; ^†^interactions between DM and IR (ANOVA).

Thirty-two rats were assigned equally to the control group, the irradiation (IR) group, the DM group, and the DM combined with irradiation (DM+IR) group in equal numbers (n=8) using a computer-generated random numbers table by a technician who was not involved in the current study. The experimental protocols of the induction of the insulin-dependent DM animal model and the application of focal radiotherapy are shown in Figure 1A. Sixteen rats were intraperitoneally injected with streptozotocin (Sigma-Aldrich, USA) dissolved in 0.1 M citrate buffer (pH: ∼4.5) with a single dose of 60 mg/kg body weight after an overnight fast to establish the type 1 DM model. The remaining 16 rats were injected with equivalent sterile citrate buffer without streptozotocin. The fasting glucose level of each rat was detected (fasting period: 7 am to 1 pm, blood collected at 1 pm) from the tail vein 72 h after streptozotocin injection using a glucometer (OneTouch SureStep Plus, Lifescan, USA), and animals with glucose levels greater than 300 mg/dL were considered to be the qualified diabetic model.

In this study, all 16 rats injected with streptozotocin had a glucose level higher than 300 mg/dL. Fasting glucose levels of all rats in each group were also measured 1, 2, and 3 months after streptozotocin injection (blood collected at 1 pm). One month after streptozotocin injection, rats in the IR and DM+IR groups were subjected to focal radiotherapy with the clinical relevant dose of 8 Gy twice, at days 1 and 3, at the distal metaphyseal area of the right femur covering a 1×1 cm square collimated field under anesthesia. Bone mass, microarchitecture, turnover, as well as bone cell activities were determined 2 months post-irradiation. All rats were subjected to intraperitoneal calcein injection (Sigma-Aldrich, 8 mg/kg) 14 and 4 days before sacrifice.

### Serum biochemical analysis

Blood samples of rats were collected from the abdominal aorta for serum biochemical analysis. After centrifugation (1500 *g*, 25°C, 5 min), the concentration of bone formation markers, including osteocalcin and procollagen type 1 N-terminal propeptide (P1NP), and bone resorption markers, including tartrate-resistant acid phosphatase 5b (TRAcP5b) and C-telopeptide of type I collagen (CTX-I), in serum were quantified using commercial enzyme-linked immunosorbent assay (ELISA) kits (CUSABIO Biotech Co., China). The entire procedure was strictly performed following the manufacturer's instruction.

### Micro-CT imaging

After sacrifice, the characteristics of the right femur of all animals were assessed with a micro-CT scanning system (GE healthcare, USA). The femoral samples were acquired and fixed in 85% ethanol for 48 h before scanning. The parameters used during micro-CT scanning were as follows: voltage of 80 kV, current of 80 μA, exposure time of 3000 ms, rotation angle of 360°. Then, all scanned two-dimensional images were reconstructed to three-dimensional volumes with 16-μm isotropic voxel size. The volume of interest (VOI) for trabecular bone analysis was manually contoured starting from the proximal end of the growth plate of the distal femur and extending 2.5 mm to the proximal end using the VG Studio Max 2.2 software (Volume Graphics, Germany). The architecture indices associated with femoral trabecular bone, including bone volume/total volume (BV/TV), bone surface/bone volume (BS/BV), bone mineral density (BMD), connectivity density (Conn.D), structure model index (SMI), trabecular thickness (Tb.Th), trabecular number (Tb.N), and trabecular separation (Tb.Sp) were subsequently quantified.

### Histology and histomorphometry

After micro-CT scanning, all femoral specimens were dehydrated in gradient ethanol and xylene for 2 h and embedded in methylmethacrylate. Then, the femoral bone samples were longitudinally sectioned to approximately 50-μm thickness using a diamond saw microtome (Leica 2500E, Leica SpA, Italy). All sections were imaged under a fluorescence microscope (LEICA DM LA, Leica Microsystems, Germany). The trabecular region (∼3 mm proximal to the distal growth plate) was chosen for calcein double-labeling analysis. The mineral apposition rate (MAR), mineralizing surface per bone surface (MS/BS), and the bone formation rate per bone surface (BFR/BS) of the femoral samples were calculated and quantified as previously described (19,20). The linearity of MAR and serum osteocalcin concentration in the four groups was also analyzed.

All tibial samples were fixed in 4% paraformaldehyde for 48 h after sacrifice and immersed in 15% EDTA solution for approximately 1 month. Then, the decalcified tibiae samples were embedded in paraffin. The 5-μm-thick sections were stained with toluidine blue to label osteoblasts and stained with tartrate resistant acid phosphatase (TRAcP) to label osteoclasts. The static histomorphometric parameters including osteoblast number/trabecular bone surface (N.OB/BS) and osteoclast number/trabecular bone surface (N.OC/BS) were quantified.

### Osteocyte survival analysis

The 5-μm-thick sections of all tibial specimens embedded in paraffin were stained with hematoxylin and eosin (H&E) to visualize the morphology of osteocytes and their lacunae. The quantitation of empty lacunae per trabecular bone surface in the H&E-stained images was analyzed. In addition, TUNEL immunofluorescence staining was also performed to determine the osteocytic apoptosis using a commercial immunostaining kit (Roche, Germany), following the manufacturers' protocols. The TUNEL-positive osteocytes in trabecular bone in the four groups were counted and analyzed using the fluorescence microscope.

### Real-time PCR assays

The mid-diaphysis of the right femur was centrifuged (2000 *g*, 25°C, 10 min) to flush out bone marrow and bone specimens and the bones were ground to powder in a mortar filled with liquid nitrogen for real-time PCR analysis. Then, the specimens were lysed using TRIzol regent that mixed with phenol and guanidine thiocyanate solution and the total RNA was extracted as described before (19,20). The cDNA was then synthesized from RNA using SuperScript III reverse transcriptase (Invitrogen, USA). Then, the quantitative real-time PCR was performed using SYBR Green PCR Master Mix (Applied Biosystems, USA) on the ABI 7300 Real-Time PCR system. GAPDH was used as the reference gene and all gene expression levels were calculated via 2^-ΔΔCt^ method. The sequences of the primers used in the study, including ALP, Runx2, Osx, Col-1, TRAP, Cathepsin K, Calcitonin receptor, Sost, and DKK1, which were designed and synthesized by Beijing AuGCT DNA-SYN Biotechnology Co., Ltd, (China), are shown in Table 1.

**Table 1:** Primer sequences used in quantitative real-time PCR assays.

Genes	Forward sequence (5′-3′)	Reverse sequence (5′-3′)
ALP	CCTAGACACAAGCACTCCCACTA	GTCAGTCAGGTTGTTCCGATTC
Runx2	TACCAGCCACCGAGACCAA	AGAGGCTGTTTGACGCCATAG
Osx	GCTGCCTACTTACCCGTCTG	GTTGCCCACTATTGCCAACT
Col-1	TCTGACTGGAAGAGCGGAGAG	GAGTGGGGAACACACAGGTCT
TRAP	ACGTATGTGGAAGCCTCTGG	CTCCCTCAGACCCATTAGGG
Cathepsin K	TGTCTGAGAACTATGGCTGTGG	ATACGGGTAACGTCTTCAGAG
Calcitonin receptor	GCTGCTCTGATTGCTTCCAT	TTGTAGTAGACGGCACGAGT
DKK1	GCCTCCGATCATCAGACGGT	GCAGGTGTGGAGCCTAGAAG
Sost	TGATGCCACAGAAATCATCC	ACGTCTTTGGTGTCATAAGG
GAPDH	ACCACAGTCCATGCCATCAC	TCCACCACCCTGTTGCTGTA

### Statistical analysis

Data analyses were performed in the SPSS 21.0 software (SPSS, USA). All data are reported as means±SD. The Kolmogorov-Smirnov test and Levene's test were used to examine the normal distribution and homoscedasticity determination, respectively. All parameters were consistent with normal distribution and homoscedasticity. Two-way ANOVA with Bonferroni's *post hoc* test was then employed to compare differences in bone mass, microarchitecture, turnover, cellular activities, and mRNA expression data between irradiated and non-irradiated samples in the rats. Two-way ANOVA was used to assess significant effects of DM and IR interaction (P<0.05) according to the indices of micro-CT (including BV/TV, BS/BV, BMD, Conn.D, SMI, Tb.Th, Tb.N, and Tb.Sp), serum biochemical levels (including osteocalcin, P1NP, TRAcP5b, and CTX-I), bone histology (including N.OB/BS and N.OC/BS), bone histomorphometry (including MAR, MS/BS, and BFR/BS), osteocyte survival (including empty lacunae and TUNEL^+^ osteocytes), and skeletal gene expression (including ALP, Runx2, Osx, Col-1, TRAP, Cathepsin K, Calcitonin receptor, Sost, and DKK1).

## Results

### Glucose metabolism

Fasting glucose levels of rats in the DM and DM+IR groups were significantly increased compared with those in the control group 1, 2, and 3 months after streptozotocin administration (P<0.001), whereas fasting glucose levels had no statistical difference between the DM and DM+IR groups at any time-point (P>0.05). Moreover, no significant difference was observed in the fasting glucose levels between the IR group and the control group at any time-point (P>0.05, Figure 1B).

### Micro-CT analysis

As shown in Figure 1C-K, representative micro-CT images revealed that femoral trabecular bone microstructure in the IR group showed deterioration compared with the control group, as evidenced by the statistically significant decrease in BV/TV (-26.9%), BMD (-28.0%), Conn.D (-31.7%), Tb.Th (-11.4%), and Tb.N (-27.0%), and increase in BS/BV (+32.5%), SMI (+41.7%), and Tb.Sp (+15.6%). DM rats also showed significantly lower BV/TV (-29.3%), BMD (-29.3%), Conn.D (-27.6%), Tb.Th (-14.3%), and Tb.N (-27.0%) and higher BS/BV (+31.5%), SMI (+50.3%), and Tb.Sp (+21.3%) than rats in control groups. Moreover, femoral trabecular bone microstructure in the DM+IR rats was more damaged compared with the IR and DM rats. The results of statistical analysis also showed that BV/TV, BMD, Conn.D, Tb.Th, and Tb.N in DM+IR rats were significantly lower than the IR rats (-22.1, -23.0, -35.0, -17.2, and -18.5%, respectively) and DM rats (-19.4, -21.7, -38.6, -14.3, and -18.5%, respectively). The BS/BV, SMI, and Tb.Sp in the DM+IR group were significantly higher than the IR group (+23.8, +28.2, and +22.2%, respectively) and the DM group (+24.7, +20.9, and +16.5%, respectively).

### Serum biochemical analysis

**Figure 2:**
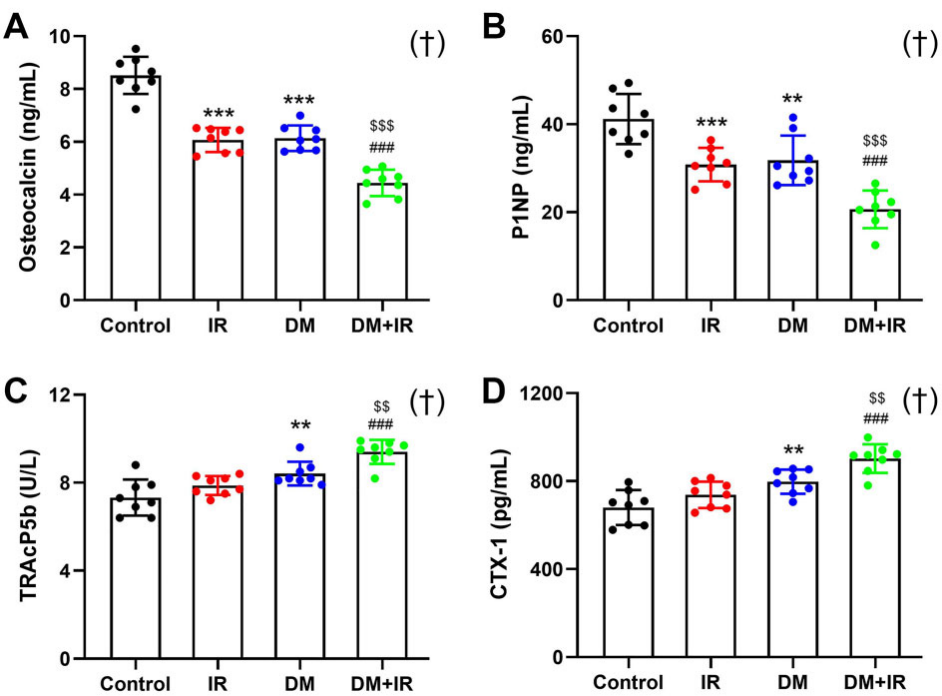
Effects of focal irradiation on serum concentrations of bone turnover markers in streptozotocin-treated diabetic rats or non-diabetic rats. **A** and **B**, Quantitative analysis of bone formation markers, including osteocalcin and N-terminal propeptide of type 1 procollagen (P1NP) in the Control, irradiation (IR), diabetes mellitus (DM), and DM+IR rats. **C** and **D**, Quantitative analysis of bone resorption markers, including tartrate-resistant acid phosphatase 5b (TRAcP5b) and C-telopeptide of type I collagen (CTX-I) in the Control, IR, DM, and DM+IR groups. Data are reported as means±SD (n=8). **P<0.01, ***P<0.001 *vs* the Control group; ^###^P<0.001 *vs* the IR group; ^$$^P<0.01, ^$$$^P<0.001 *vs* the DM group; ^†^interactions between DM and IR (ANOVA).

The results of serum biochemical analysis via ELISA assays are shown in Figure 2. The concentrations of osteocalcin and P1NP, the bone formation markers in serum, were significantly decreased by 28.8 and 25.1% in IR rats compared with the control rats (P<0.001). Rats in the DM group also showed a significant decrease in osteocalcin (-27.9%, P<0.001) and P1NP (-22.8%, P<0.01) compared with the control rats. However, the concentrations of serum osteocalcin and P1NP in DM+IR rats were further suppressed compared with the IR rats (-26.9%, P<0.001; -32.9%, P<0.001, respectively) and DM rats (-27.7%, P<0.001; -35.0%, P<0.001, respectively). In addition, the concentrations of serum TRAcP5b and CTX-I (the bone resorption markers) were significantly increased in DM rats (+14.7%, P<0.01; +17.4%, P<0.01, respectively) compared with the control rats. The IR rats exhibited no observable difference in serum TRAcP5b or CTX-I secretion compared with the control rats. However, the TRAcP5b and CTX-I concentrations in DM+IR rats were significantly increased compared with the IR rats (+19.3%, P<0.001; +22.4%, P<0.001, respectively) and DM rats (+11.8%, P<0.01; +13.2%, P<0.01, respectively).

### Histology and histomorphometry analyses

**Figure 3:**
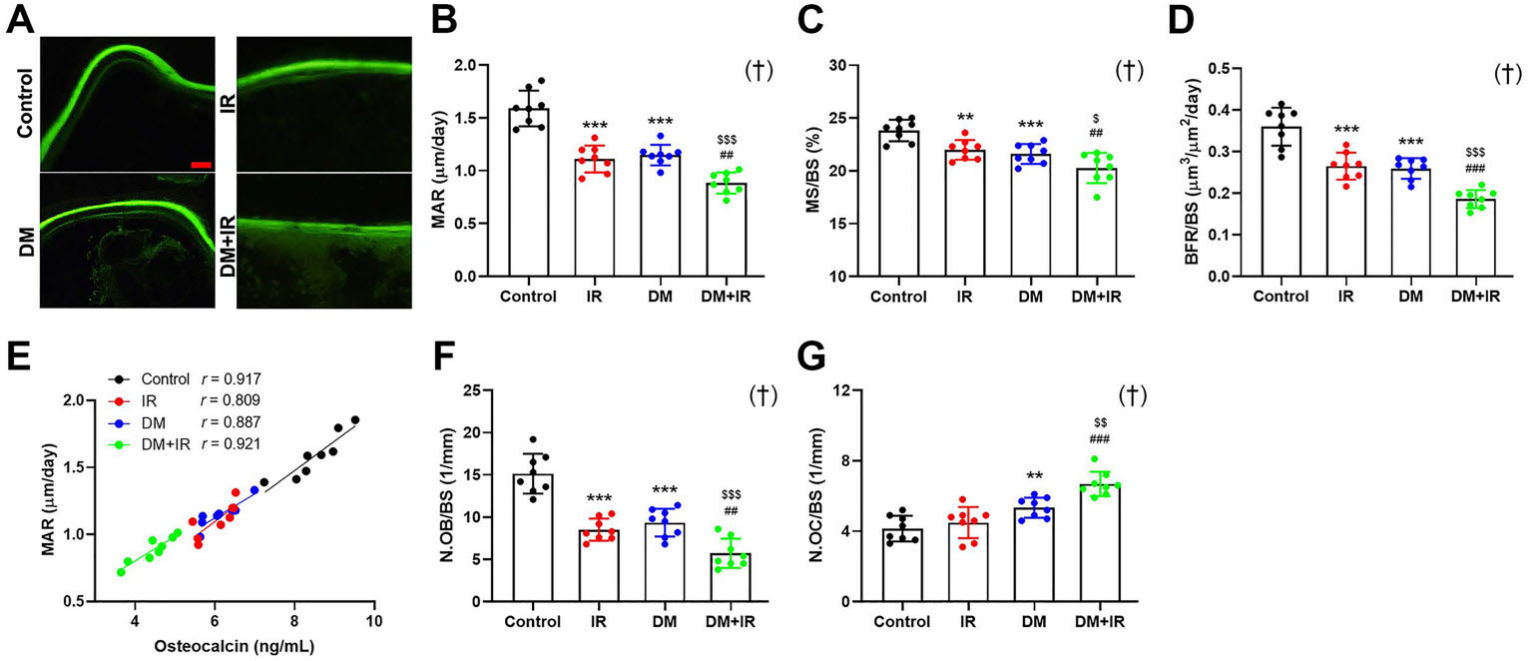
Effects of focal irradiation on bone histological and histomorphometric parameters in streptozotocin-treated diabetic rats or non-diabetic rats. **A**, Representative calcein double labeling images of the femoral trabecular bone in the Control, irradiation (IR), diabetes mellitus (DM), and DM+IR rats. Scale bar 20 µm. **B-D**, Quantitative analysis of dynamic histomorphometry for mineral apposition rate (MAR), mineralizing surface per bone surface (MS/BS), and bone formation rate per bone surface (BFR/BS). **E**, Linear regression analysis of MAR and concentration of osteocalcin in serum. **F** and **G**, Static histomorphometric analysis for number of osteoblasts (N.OB/BS) and osteoclasts (N.OC/BS) on femoral trabecular bone in the control, IR, DM, and DM+IR groups. Data are reported as means±SD (n=8). **P<0.01, ***P<0.001 *vs* the Control group; ^##^P<0.01, ^###^P<0.001 *vs* the IR group; ^$^P<0.05, ^$$^P<0.01, ^$$$^P<0.001 *vs* the DM group; ^†^interactions between DM and IR (ANOVA).

According to the dual calcein labeling results (Figure 3A-D), the MAR (-31.3%, P<0.001), MS/BS (-7.7%, P<0.01), and BFR/BS (-24.3%, P<0.001) in the IR rats were significantly decreased compared with the control rats. The DM rats also showed significantly lower MAR (-29.8%), MS/BS (-9.4%), and BFR/BS (-27.1%) than the control rats (P<0.001). The MAR, MS/BS, and BFR/BS in the DM+IR rats were further decreased compared with the IR rats (-18.2%, P<0.01; -7.8%, P<0.01; -32.1%, P<0.001, respectively) and DM rats (-26.2%, P<0.001; -6.1%, P<0.05; -29.8%, P<0.001, respectively). The fitted curve between the MAR of the femoral samples and the concentration of serum osteocalcin in control, IR, DM, and DM+IR rats exhibited a high linear relation as shown in Figure 3E.

The histological results (Figure 3F and G) showed that the IR and DM groups exhibited significantly lower N.OB/BS than the control rats (-43.3%, P<0.001; -36.6%, P<0.001, respectively). The N.OB/BS in the DM+IR group was significantly decreased by 44.1% (P<0.01) and 47.3% (P<0.001) compared with the IR and DM groups, respectively. Moreover, N.OC/BS in the DM group was significantly increased by 39.9% (P<0.01) compared to the control group. DM plus irradiation led to a further increase in N.OC/BS compared with the IR (+49.8%, P<0.001) and DM (+23.5%, P<0.01) rats.

### Osteocyte survival analysis

**Figure 4:**
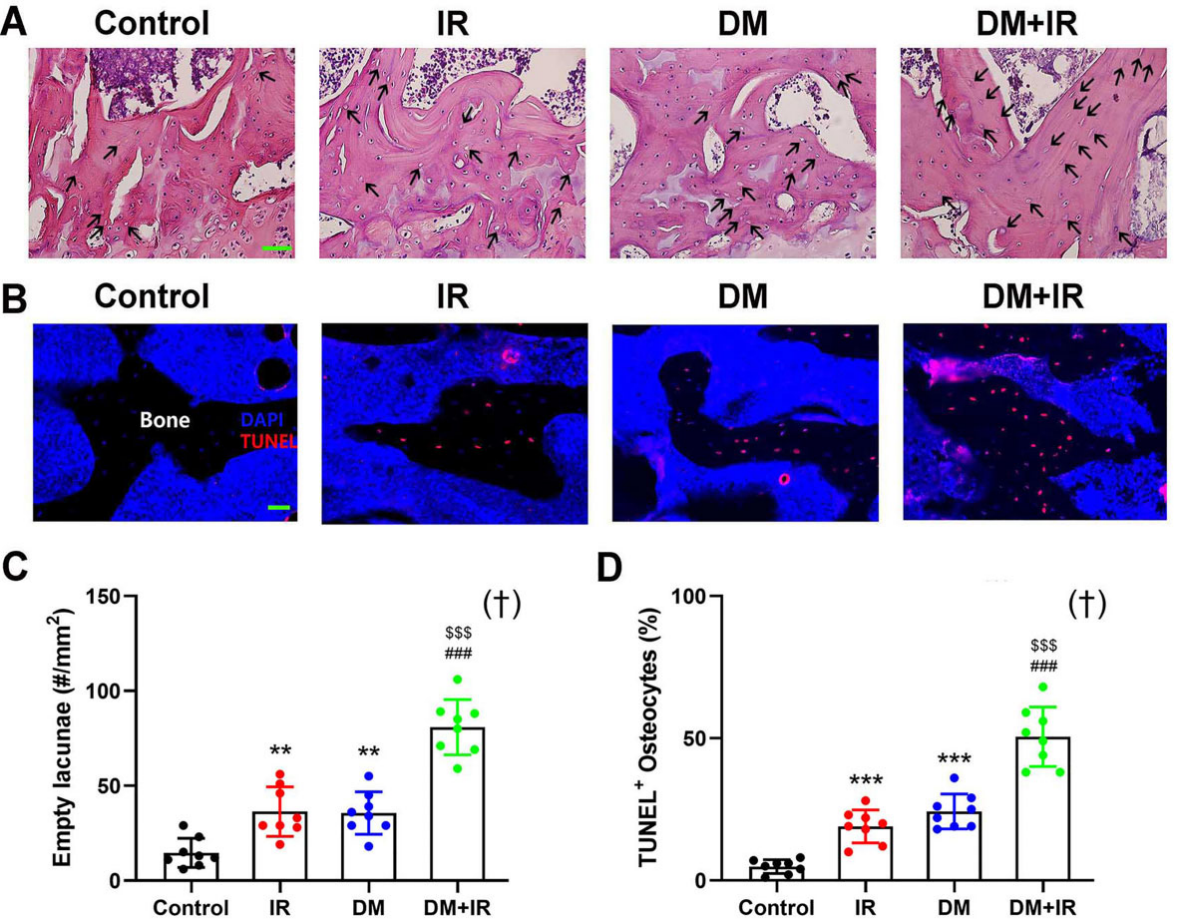
Effects of focal irradiation on the survival and activity of osteocytes in femoral trabeculae in streptozotocin-treated diabetic rats or non-diabetic rats. **A** and **B**, Representative H&E and TUNEL immunofluorescence staining images performed on decalcified tibial specimens. Empty lacunae are indicated with black arrows. **C** and **D**, Quantitative analysis of empty lacunae based on the H&E staining and TUNEL-positive osteocytes per trabecular bone surface in the Control, irradiation (IR), diabetes mellitus (DM), and DM+IR rats. Scale bar 100 µm. Data are reported as means±SD (n=8). **P<0.01, ***P<0.001 *vs* the Control group; ^###^P<0.001 *vs* the IR group; ^$$$^P<0.001 *vs* the DM group; ^†^interactions between DM and IR (ANOVA).

The results of H&E staining (Figure 4A and C) demonstrated that the tibial samples of rats in IR and DM groups had significantly higher number of empty lacunae osteocytes than the control group (P<0.01). The number of empty osteocytic lacunae in DM+IR rats was further significantly increased compared with the IR and DM rats (P<0.001). In addition, the TUNEL staining results (Figure 4B and D) revealed that IR and DM rats showed significantly increased apoptotic TUNEL+ osteocyte number compared with the rats in the control group (P<0.001). The TUNEL+ osteocyte number in the tibial samples of rats in the DM+IR group was further significantly higher than the IR and DM groups (P<0.001).

### Skeletal gene expression analysis

**Figure 5:**
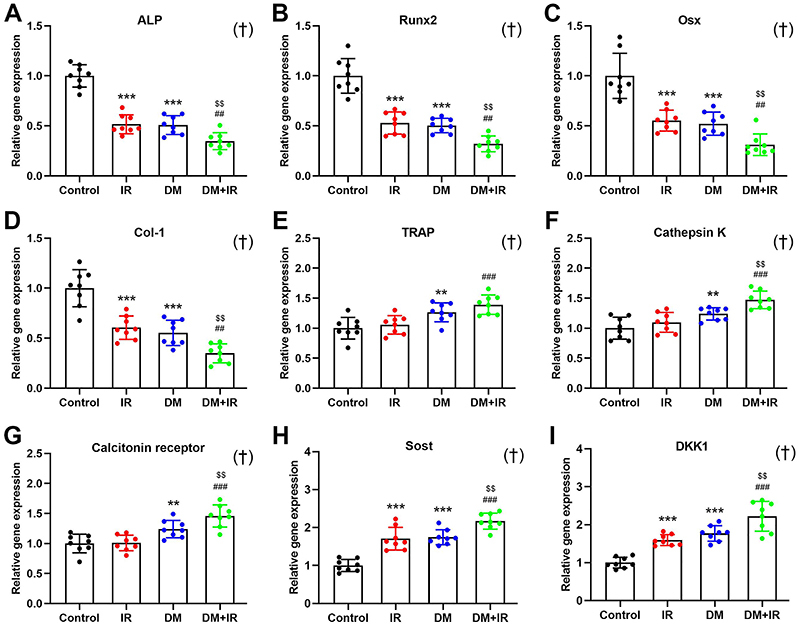
Effects of focal irradiation on skeletal relative gene expression levels of (**A**-**D**) osteoblast-related markers (ALP, Runx2, Osx, and Col-1), (**E-G**) osteoclast-related markers (TRAP, Cathepsin K, and Calcitonin receptor), and (**H-I**) Wnt inhibitors (Sost and DKK1) in the mid-diaphysis of the right femur in streptozotocin-treated diabetic rats and non-diabetic rats. Data are reported as means±SD (n=8). **P<0.01, ***P<0.001 *vs* the Control group; ^##^P<0.01, ^###^P<0.001 *vs* the IR (irradiated) group; ^$$^P<0.01 *vs* the DM (diabetic) group; ^†^interactions between DM and IR (ANOVA).

As shown in Figure 5A-D, both IR and DM significantly suppressed the relative gene expression levels of osteoblast-specific markers, including ALP, Runx2, Osx, and Col-1 (P<0.001). Rats in the DM+IR group exhibited much lower ALP, Runx2, Osx, and Col-1 expression than IR and DM rats (P<0.01). DM, but not IR, significantly increased the osteoclast-related TRAP, Cathepsin K, and Calcitonin receptor gene expression (Figure 5E-G) compared with control rats (P<0.01). Moreover, the gene expression levels of osteoclast-related markers of rats in the DM+IR group were further significantly increased compared to rats in the IR (P<0.001) and DM (P<0.05) groups. As shown in Figure 5H and I, gene expression levels of Sost and DKK1 were significantly higher in both IR and DM rats than in control rats (P<0.001). DM+IR caused further up-regulation of gene expression of Sost and DKK1 compared with IR (P<0.001) and DM (P<0.01) rats, respectively.

## Discussion

A growing body of evidence suggests that both DM and radiotherapy can significantly suppress bone quantity and quality and impair bone homeostasis (21-23). However, the bone phenotype of the diabetic patient or animal subjected to irradiation exposure has never been systematically characterized either clinically or experimentally. In this study, we observed that streptozotocin-induced DM rats exhibited more significant decrease in bone mass and more significant deterioration in trabecular bone microarchitecture following radiotherapy compared with DM or irradiation alone. Furthermore, our serum biochemical, bone histomorphometric, and real-time PCR results demonstrated that DM combined with irradiation induced a more pronounced decrease in osteoblast number and differentiation and bone formation compared with DM or irradiation alone; nonetheless, DM rats exhibited more notable increases in osteoclast number and bone resorbing capacity following radiotherapy. Moreover, a decrease was found in the number and survival of osteocytes and the Sost and DKK1 expression in DM rats with radiotherapy.

Our micro-CT results demonstrated that 3 months after a single high-dose streptozotocin injection DM rats exhibited significant deterioration in femoral trabecular bone microstructure compared with control rats, as evidenced by decreased BV/TV, BMD, Conn.D, Tb.Th, and Tb.N. and increased BS/BV, SMI, and Tb.Sp. Similarly, previous studies have also revealed compromised cancellous bone microarchitecture in insulin-dependent diabetic patients and animals based on HR-pQCT and micro-CT imaging (24,25). Moreover, we found that femoral trabeculae of rats showed notable disruption of cancellous bone microarchitecture and loss of trabecular elements 2 months after radiotherapy, which was consistent with previous *in vivo* findings (9,26). Interestingly, following radiotherapy DM rats had much lower volume of calcified bone tissues than DM or irradiation treatment, as evidenced by lower BV/TV and BMD, and higher BS/BV. DM plus irradiation rats also had significantly lower Conn.D, Th.Th, and Tb.N, and higher SMI and Tb.Sp, revealing much poorer trabecular connectivity and higher proportion of rod-like trabecular network. Thus, our results revealed for the first time that the diabetic rats had further aggravation of bone damage following radiotherapy, and suggested that attention should be paid to this issue in clinics and that measures against bone deterioration and fragility fractures are highly necessary.

We further investigated bone turnover and bone cell activities to identify the potential etiology of bone deterioration induced by DM combined with radiotherapy. Our results of circulating markers (serum OCN and P1NP) and tissue-level dynamic histomorphometry demonstrated that both DM and irradiation led to negative impacts on bone formation rate. In contrast, no significant change of bone resorption was found in radiation rats, and an only a mild increase in bone resorption was observed in DM rats. Growing evidence has substantiated that suppressed bone formation capacity is the major contributor to DM-induced or radiotherapy-induced bone loss (25,27,28). Furthermore, we found that DM rats exhibited much lower bone formation rate following radiation than single DM or radiotherapy alone. Interestingly, significantly higher bone resorption rate was observed in rats with DM plus irradiation than either intervention alone. Thus, our findings suggested that both therapies remarkably decreased bone formation and increased bone resorption.

At the cellular level, our static bone histomorphometric and real-time PCR results demonstrated that the number of osteoblasts as well as their differentiation capacity in rats with DM or irradiation were compromised compared with control rats. The damage to osteoblast growth and differentiation induced by DM or radiotherapy has also been reported by previous *in vitro* and *in vivo* data (29-31). Similar with the serum results for bone resorption, rats with irradiation exhibited changes in osteoclast number and osteoclast-related marker expressions, and DM induced a mild increase in osteoclast number. Moreover, DM plus irradiation led to further reduction of osteoblast number and expression of osteoblast gene markers, revealing the additive damage to osteoblasts upon combination. Surprisingly, rats exposed to DM combined with irradiation also displayed much higher osteoclast populations in bone than rats with DM or irradiation alone. Our findings indicated that the combination of DM and irradiation induced further inhibition of osteoblast activity/function and stimulation of osteoclast activity, and consequently impaired bone homeostasis and aggravated bone loss.

Osteocytes, as the most abundant and long-lived cells in adult skeleton, are regarded as important regulators of bone homeostasis (32,33). Osteocytes can directly mediate phosphate and calcium metabolism and send regulative factors to distant organs (e.g., kidney and parathyroid) (34-36). More importantly, osteocytes are found to be a powerful mechanosensor and mechanotransducer, which can orchestrate bone remodeling by the secretion of cytokines though their long dendritic processes to regulate osteoblast and osteoclast activities (e.g., sclerostin and RANKL) (37). Several previous studies have found that high glucose exposure decreases osteocyte viability and increases sclerostin expression (a negative regulator of bone formation) (38,39). Similarly, radiation has also been reported to impair osteocyte survival in rodents (40). Our findings demonstrated that both DM and irradiation increased osteocyte apoptosis and also stimulated osteocyte-specific Sost gene expression. Moreover, DM rats with radiotherapy exhibited much lower osteocyte survival and higher Sost expression than DM or IR alone. Our findings suggested that osteocyte damage was also implicated in bone deterioration induced by DM combined with radiotherapy. Furthermore, DKK1, another important negative molecule of Wnt signaling, was also found significantly higher in the skeleton of DM rats with radiotherapy than either DM or IR. Thus, our results also indicated that bone damage induced by DM combined with radiotherapy may also be associated with the increased expression of Wnt signaling modulators DKK1 and Sost in bone cells.

This study not only helps enrich basic knowledge on radiotherapy-mediated bone complications, but also raises needed attention in clinics to bone issues in diabetic patients following radiotherapy. Thus, it will be interesting and important to identify potential therapeutic options for resisting bone deterioration induced by DM plus radiotherapy in future studies.
